# Supplementary figures and images for: Precision phenotyping of a barley diversity set reveals distinct drought response strategies
Source: Front Plant Sci. 2024 Jun 24;15:1393991. doi: 10.3389/fpls.2024.1393991 (PMC11231632; doi:10.3389/fpls.2024.1393991)

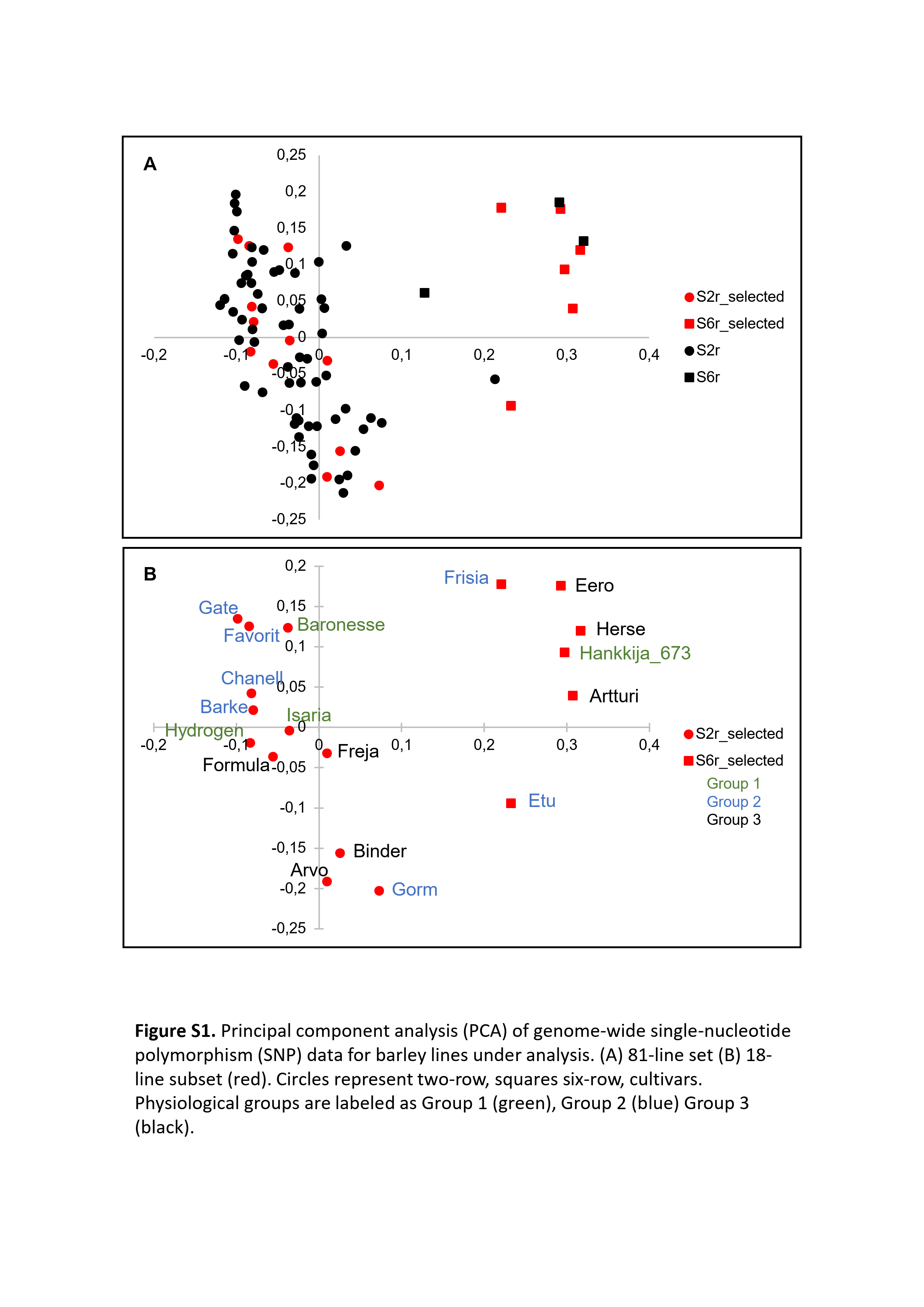

Supplement: Supplementary Figure 1 — Principal Components Analysis (PCA) plot of barley diversity set. [file Image_1.tif]

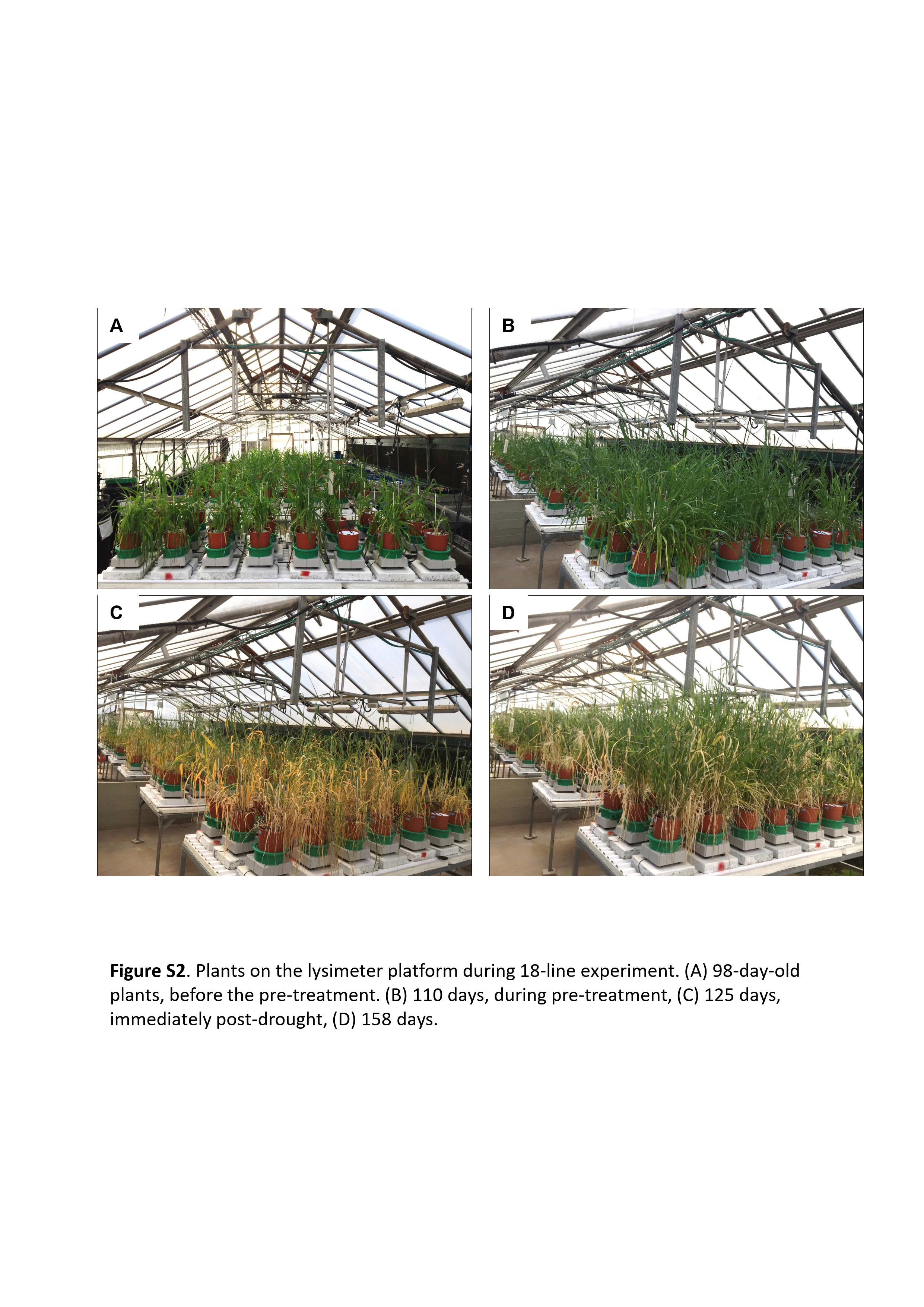

Supplement: Supplementary Figure 2 — Plants on the lysimeter platform during 18-line experiment. [file Image_2.tif]

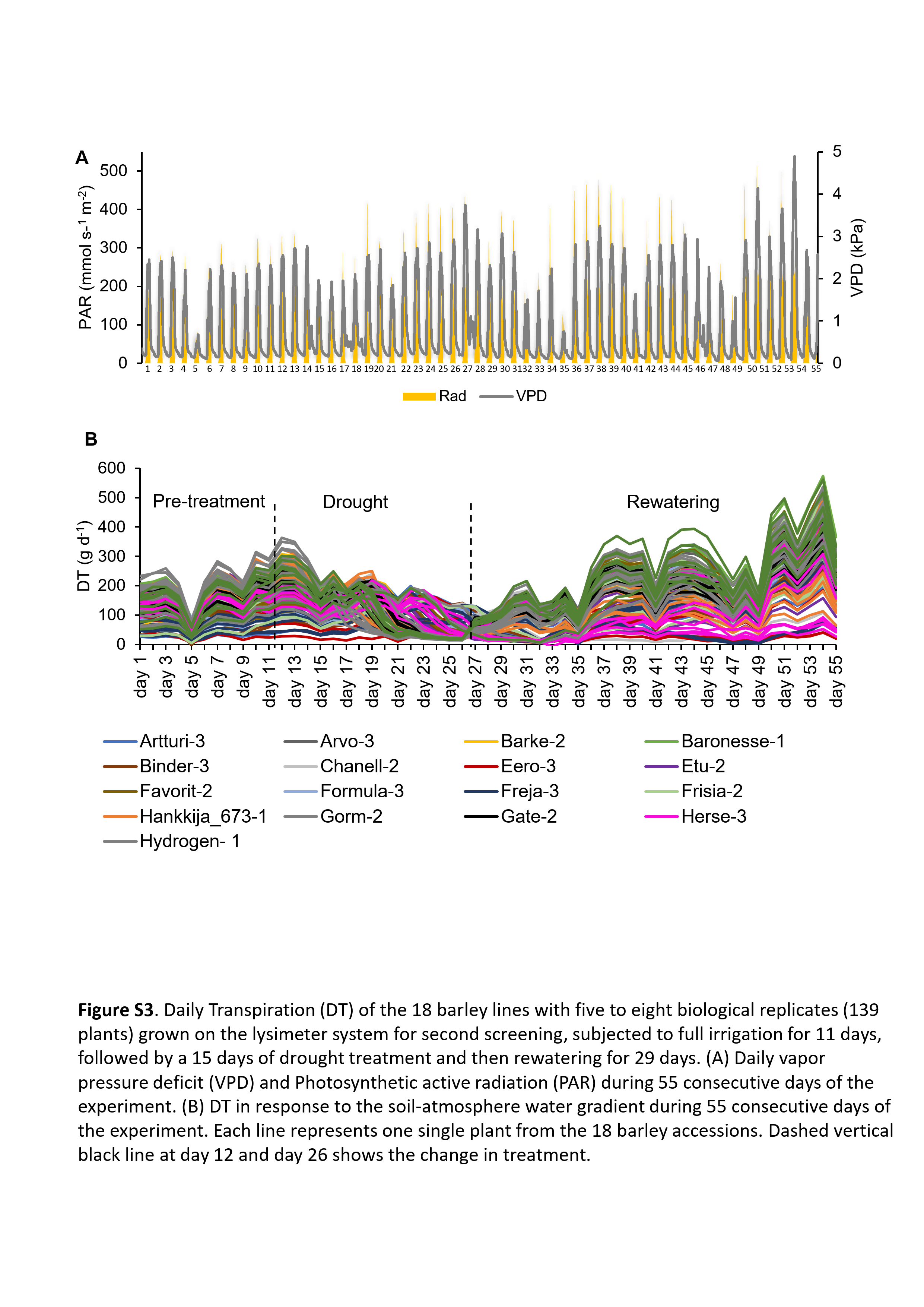

Supplement: Supplementary Figure 3 — Daily Transpiration (DT) of the 18 barley lines. [file Image_3.tif]

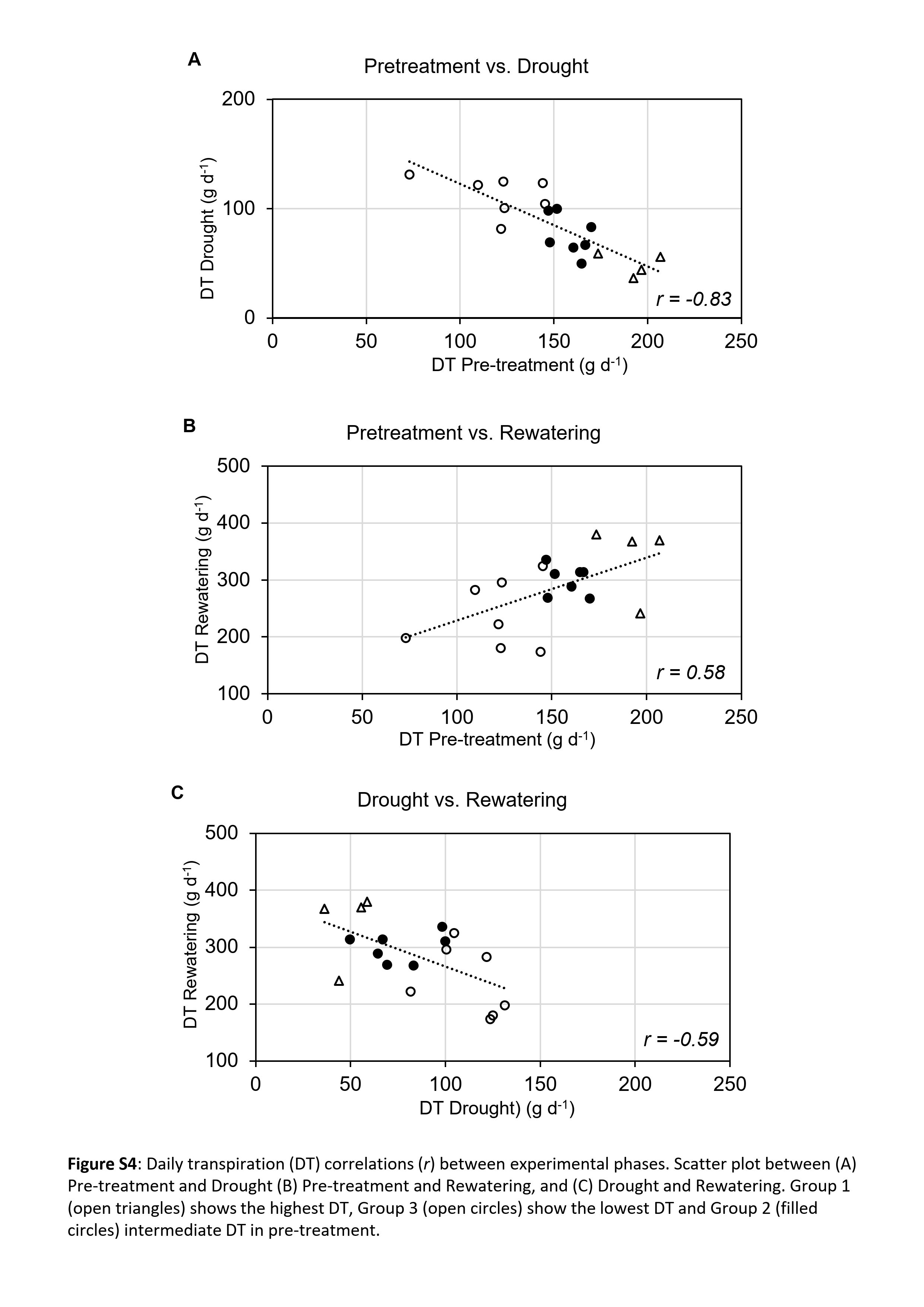

Supplement: Supplementary Figure 4 — Daily transpiration (DT) correlations (r) between experimental phases. [file Image_4.tif]

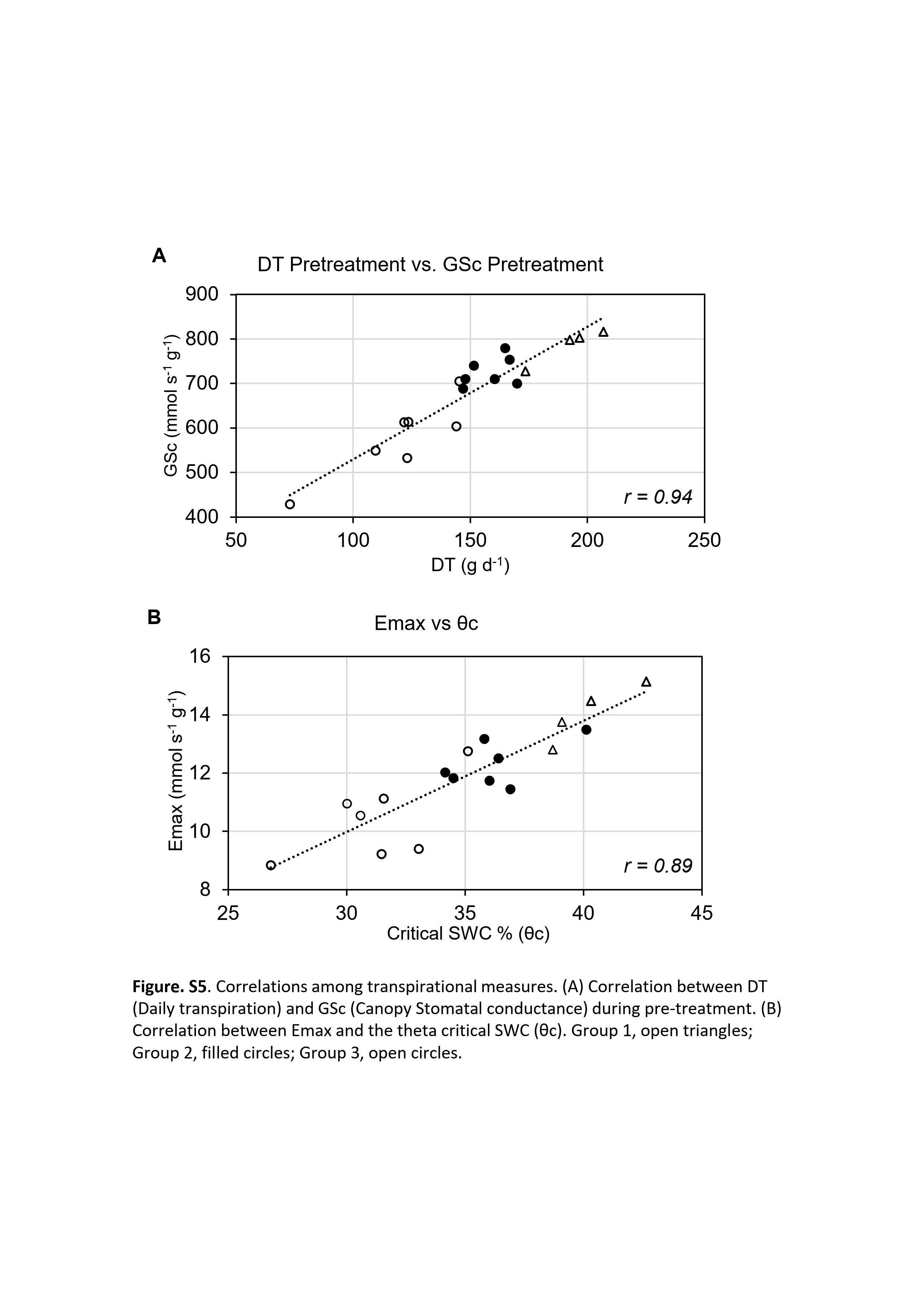

Supplement: Supplementary Figure 5 — Correlations among transpirational measures. [file Image_5.tif]

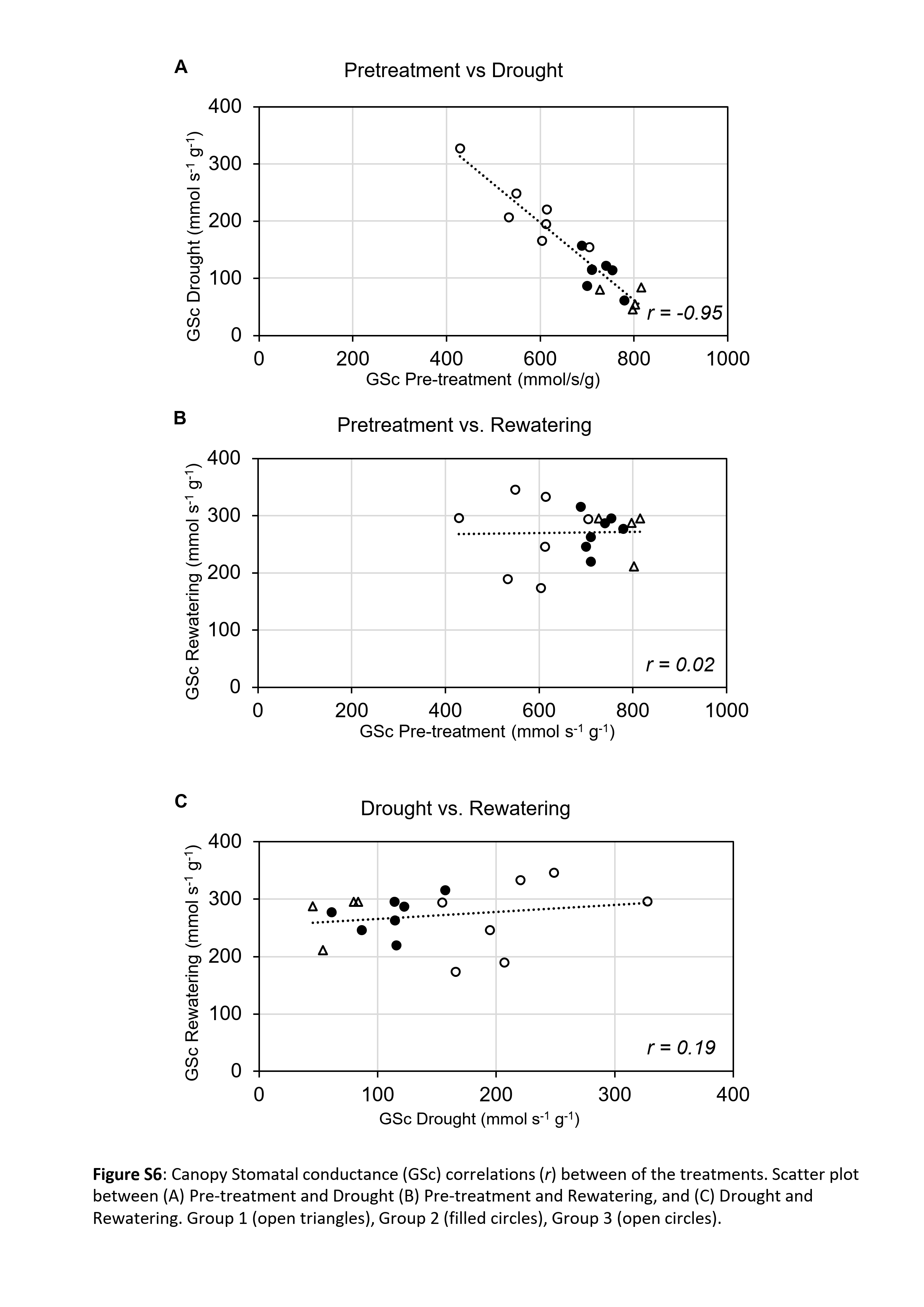

Supplement: Supplementary Figure 6 — Canopy Stomatal conductance (GSc) correlations (r) between of the treatments. [file Image_6.tif]

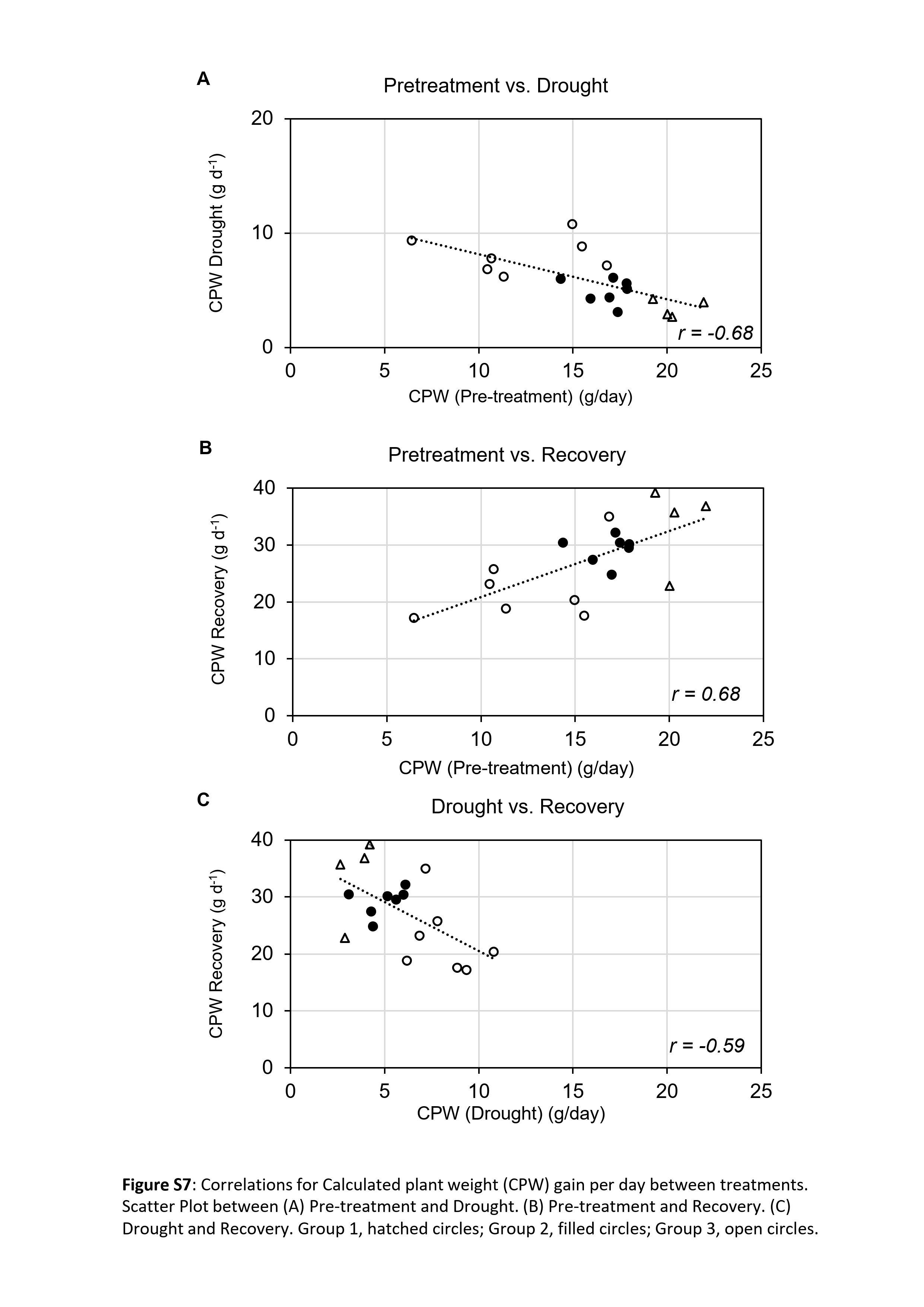

Supplement: Supplementary Figure 7 — Correlations for Calculated plant weight (CPW) gain per day between treatments. [file Image_7.tif]

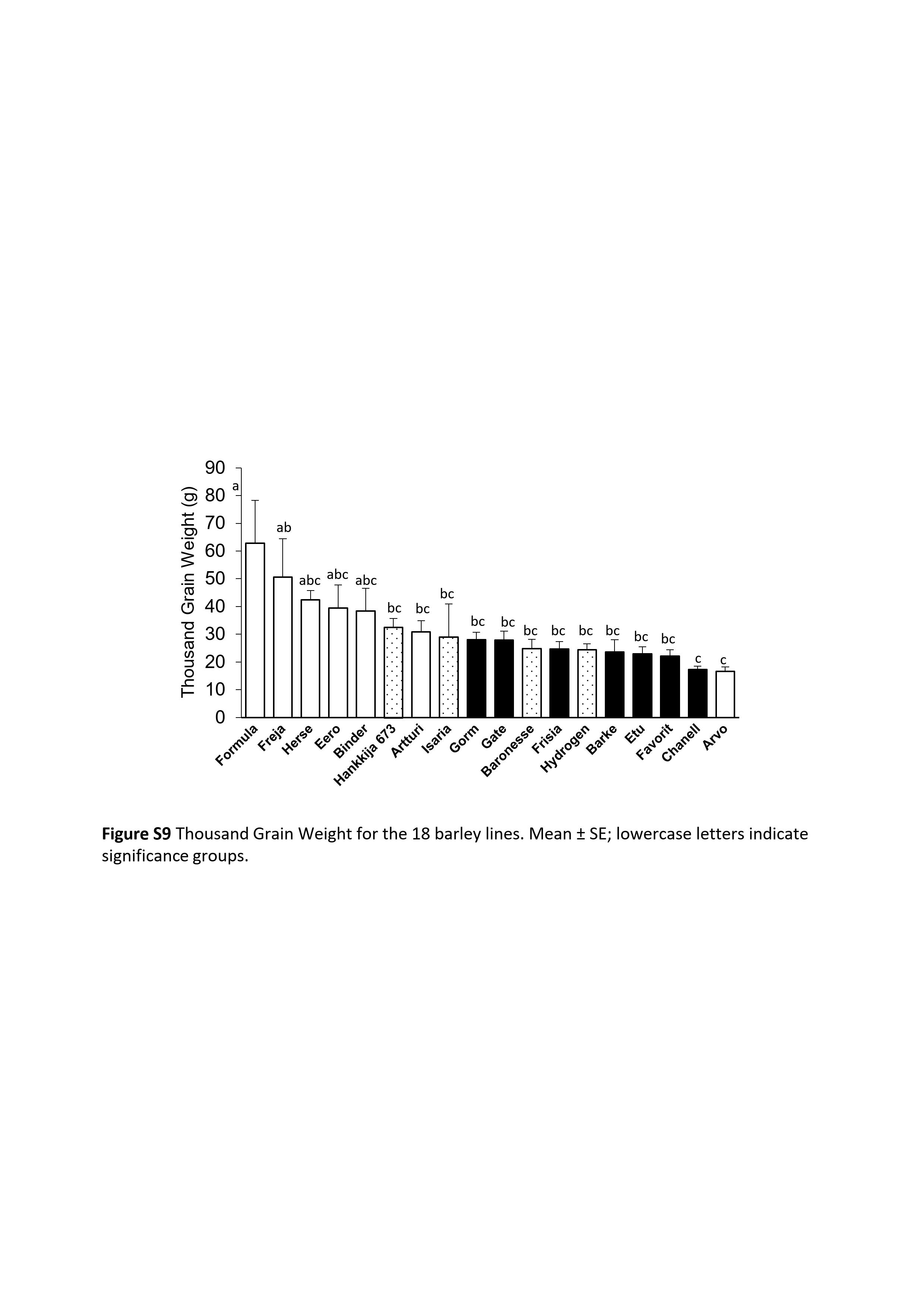

Supplement: Supplementary Figure 9 — Thousand Grain Weight for the 18 barley lines. [file Image_9.tif]
