# Supplementary material for: Precision phenotyping of a barley diversity set reveals distinct drought response strategies
Source: Front Plant Sci. 2024 Jun 24;15:1393991. doi: 10.3389/fpls.2024.1393991 (PMC11231632; doi:10.3389/fpls.2024.1393991)
Supplement: Supplementary file 10 [file Table_1.pdf]

Table S1. 81 spring barley lines for first screening

Accession	Code	Name	Rows	Release	Country	Pedigree	Breeder
126	2009	Aapo	2	1975	Finland	Carlsberg x Riegel	Hankkija Plant Breeding Institute
128	2010	Abava	2	1978	Latvia	Mari/Elsa//Domen	Stende Plant Breeding Station
129	2011	Agneta	6	1981	Sweden	Asa x Frisia x EddaII x Monte Christo	Svalöf
131	2012	Akcent	2	1992	Czech Republic	Salome/EP 79	Selgen Stupice
132	2013	Akka	2	1970	Sweden	(Arla)6 x Monte Cristo	Weibull
135	2015	Alis	2	1985	Denmark	Triumpf x Rosie Abed	Abed
136	2016	Alliot	2	1999	Denmark	Chariot x Alexis	Pajbjergfonden
137	2018	Anni	2	1980	Estonia	Lola x Liisa	Jogeva Plant Breeding Institute
142	2019	Ansis	2	1996	Latvia	Jarek/Taifun	Stende Plant Breeding Station
143	2020	Apex	2	1984	Netherlands	Aramir x F1[CEB 6721 x (Julia3 x Volla x L100)]	Cebeco
144	2021	Aramir	2	1974	Netherlands	Volla x Emir	Cebeco
148	2024	Artturi	6	1992	Finland	Arra x Nord	Boreal Plant Breeding Ltd
149	2025	Arvo	2	1966	Finland	Balder x Helmi	Agricultural Experiment Station of Finland, Jokioinen
153	2027	Athos	2	1975	France	207*Emir	Desprez
154	2028	Atlas	2	1976	Czech Republic	M- SS 55/Diamant	Selgen Stupice
155	2029	Atribut	2	1996	Czech Republic	KM V 3-83/BR 2174	Selgen Stupice
158	2030	Balder J	2	1964	Sweden	Balder X-Ray Mutant	Weibull
159	2031	Balga	2	1990	Latvia	Gunilla/KM 1192	APP Valsts Priekulu laukaugu selekcijas instituts
160	2032	Barabas	2	2005	Denmark	SJ 970621 x (Lux x Annabell)	Sejet
162	2033	Barke	2	1996	Germany	Libelle x Alexis	Breun
163	2034	Baronesse	2	1989	Germany	((343/6 x V34/6) x J -427) x (Oriol x LBW6153 P40)	Nordsaat
164	2035	Beatrix	2	2004	Germany	Viskosa x Pasadena	Nordsaat
165	2036	Berenice	2	1972	France	Union*((Bordia*Kenia)*Frisia)	INRA
166	2037	Binder	2	1916	Denmark	HOR3684/76 ABED selection in Hanna	Abed
168	2039	Birka	2	1981	Sweden	Baladi16 x (Rika x ((Tellus x (Monte_Christo x Tellus MD))	Weibull
169	2040	Blenheim	2	1992	United Kingdom	Triumph*Egmont	PBI
173	2043	Braemar	2	2000	United Kingdom	NFC 5563 x NFC 94.20	New Farm Crops (NFC)
174	2044	Brazil	2	2001	France	Trebon x Cooper	Momont
176	2045	Britta A	2	1964	Sweden	(Binder*Opal)*(Balder*Kenia)	Lantmännen SW Seed
180	2046	Caja	2	1980	Denmark	PF-M-13 x PF 62-6/6-4	Pajbjerg
187	2048	Carlsberg II	2	1947	Denmark	Prentice x Maja	Carlsberg
190	2050	Ceylon	2	2003	Netherlands	Portia x Amber	Cebeco
193	2051	Chanell	2	2006	Denmark	Barke x Ca 500201	Carlsberg
194	2052	Chariot	2	1992	United Kingdom	Dera*(Carnival*Atem)	PBI
200	2053	Claret	2	1980	United Kingdom	((Proctor*HP 5466)*Armelle)*Abacus	Nickerson
201	2054	Class	2	2003	United Kingdom	Prestige x Optic	PBI
205	2056	Cooper	2	1994	United Kingdom	(Corniche*Force)*Troop	New Farm Crops (NFC)
206	2057	Corgi	2	1983	United Kingdom	Triumph*15533 Co	Welsh Pl Breed Stn
210	2059	Dandy	2	1988	United Kingdom	Egmont*Atem	Welsh Pl Breed Stn
209	2060	Croydon	2	1982	Sweden	welam x tellus M_1D	Weibull
208	2061	Cristalia	2	2004	United Kingdom	Ortoli x Brise	Syngenta
211	2062	Danuta	2	2000	Germany	90014DH [Krona] x (Salome x Maresi)	Nordsaat
212	2063	Deba Abed	2	1965	Denmark	Abed Denso*Weihenstephaner MR 2	Abed
213	2064	Delta	2	1959	Netherlands	Tyra*Claret OR Kenia*H.laevigatum*Gull	Cebeco
214	2065	Derkado	2	1992	Germany	Lada*Salome	Hadmersleben
215	2066	Dialog	2	2000	Denmark	Otira x (Ferment x Mentor)	Sejet
216	2067	Diamant	2	1965	Czech Republic	Valticky X-ray Mutant	OGZ
217	2068	Digersano	2	1991	Italy	Mari-Coho x Sul-Mackta	Cermis/ENEA
223	2070	Drost	2	1954	Denmark	Maja x Kenia	Pajbjerg

DETAILS	
Year	1915-2006
2- row	72
6- row	9
Breeders	38
Austria	1
Czech Republic	9
Denmark	14
Estonia	2
Finland	7
France	3
Germany	9
Italy	1
Latvia	5
Netherlands	5
Norway	1
Slovak Republic	1
Sweden	12
United Kingdon	11
14 countries	81

224	2071	Druvis	6	1999	Latvia	Dobrij/HVS 115440	Stende Plant Breeding Station
226	2072	Edda	6	1949	Sweden	Vega x Asplund	Svalöf
227	2073	Eero	6	1975	Finland	Mari 2r x Otra	Hankkija Plant Breeding Institute
228	2074	Egmont	2	1980	United Kingdom	(Maris Yak*W 1001)*Vada	PBI
229	2075	Elantra	2	1998	Denmark	Caminant x Heron	Sejet
230	2076	Elo	2	1989	Estonia	Triumph x Lofa	Jogeva Plant Breeding Institute
231	2077	Emir	2	1962	Netherlands	Delta*(Agio*(Kenia)2*Arabian Variety)	Cebeco
234	2079	Etu	6	1970	Finland	Bonus M x Varde	Boreal Plant Breeding Ltd
235	2080	Eunova	2	2000	Austria	(Serva x ML502) x CF 79	Probstdorf
237	2081	Famin	2	1996	Czech Republic	Akcent/CE 597	Hrubcice
238	2082	Favorit	2	1973	Czech Republic	Diamant/F.Union	Hrubcice
239	2083	Felicitas	2	2002	Germany	(Baronesse x Meltan) x Krona)	Breun
240	2084	Formula	2	1987	Sweden	Triumph x A 11 3109	Weibull
241	2085	Forum	2	1993	Czech Republic	H 387-75/Horpatsi Ketscoros//044-78	HYBRITECH
242	2086	Freja	2	1941	Sweden	Victory X Opal	Svalöf
243	2087	Frisia	6	1955	Germany	(Granat*Pirthgjarn)*(Eckendorfer WG*Kalckreuthen WG)	Breustedt
245	2088	Galan	2	1990	Slovak Republic	Complex hybrid. K 2567/HE 1428	Sladkovicovo
267	2089	Helmi	2	1942	Finland	Binder X Pikkio	na
268	2090	Heris	2	1998	Czech Republic	HE 4431/CE 431	Hrubcice
51	2091	Harry	2	1978	Sweden	Maythorpe Gamma-Ray Mutant	Weibull
260	2092	Hanna	2	1992	Sweden	(Armelle*Lud)*Luke	Weibull
259	2093	Hankkija_673	6	1973	Finland	(Herta 8 x Byg 191 x Ingrid x Minerva) x Kristina	Hankkija Plant Breeding Institute
258	2094	Hanka	2	1997	Germany	Swedish (Gotland) Land Variety	Semundo
257	2095	Hana	2	1973	Czech Republic	Diamant/Alsa	OGZ
255	2096	Gull	2	1915	Sweden	Swedish Land Variety	Svalöf
254	2097	Gorm	2	1981	Denmark	Otra x Paavo	Sejet
253	2098	Golf	2	1983	United Kingdom	[(monte cristo x 5690) x 5793^2] x 5853^3	Nickerson
250	2100	Gizmo	2	2006	Denmark	Prestige x Ca 800602	Carlsberg
247	2101	Gate	2	1995	Latvia	Emir/2∗Nadja//HE-497/Hadmersleben 70197/70	Priekuli
269	2102	Herse	6	1939	Norway	Asplund x Maskin	Vollebekk
271	2103	Hydrogen	2	1999	Denmark	(Alis x Digger) x Derkado	Nordic seed
283	2104	Isaria	2	1924	Germany	Bavaria x Danubia	Ackermann
