# Supplementary material for: Precision phenotyping of a barley diversity set reveals distinct drought response strategies
Source: Front Plant Sci. 2024 Jun 24;15:1393991. doi: 10.3389/fpls.2024.1393991 (PMC11231632; doi:10.3389/fpls.2024.1393991)
Supplement: Supplementary file 11 [file Table_2.pdf]

Table S2. Characteristics selected 18 spring barley lines

Accession	Code	Name	Rows	Release	Country	Pedigree	Breeder
148	2024	Artturi	6	1992	Finland	Arra x Nord	Boreal Plant Breeding Ltd
227	2073	Eero	6	1975	Finland	Mari 2r x Otra	Hankkija Plant Breeding Institute
243	2087	Frisia	6	1955	Germany	(Granat*Pirthgjarn)*(Eckendorfer WG*Kalckreuthen \	Breustedt
193	2051	Chanell	2	2006	Denmark	Barke x Ca 500201	Carlsberg
234	2079	Etu	6	1970	Finland	Bonus M x Varde	Boreal Plant Breeding Ltd
238	2082	Favorit	2	1973	Czech Re|	Diamant/F.Union	Hrubcice
259	2093	Hankkija_673	6	1973	Finland	(Herta 8 x Byg 191 x Ingrid x Minerva) x Kristina	Hankkija Plant Breeding Institute
269	2102	Herse	6	1939	Norway	Asplund x Maskin	Vollebekk
242	2086	Freja	2	1941	Sweden	Victory X Opal	Svalöf
254	2097	Gorm	2	1981	Denmark	Otra x Paavo	Sejet
247	2101	Gate	2	1995	Latvia	Emir/2*Nadja//HE-497/Hadmersleben 70197/70	Priekuli
271	2103	Hydrogen	2	1999	Denmark	(Alis x Digger) x Derkado	Nordic Seed
283	2104	Isaria	2	1939	Germany	Bavaria x Danubia	Ackermann
149	2025	Arvo	2	1966	Finland	Balder x Helmi	Agricultural Experiment Station of Finland, Jokioinen
162	2033	Barke	2	1996	Germany	Libelle x Alexis	Breun
163	2034	Baronesse	2	1989	Germany	((343/6 x V34/6) x J -427) x (Oriol x LBW6153 P40)	Nordsaat
166	2037	Binder	2	1916	Denmark	HOR3684/76 ABED selection in Hanna	Abed
240	2084	Formula	2	1987	Sweden	Triumph x A 11 3109	Weibull

DETAILS	
2- rows	12
6- rows	6
Year	1916- 2006
Breeders	16
Czech Repu	1
Denmark	4
Finland	5
Germany	4
Latvia	1
Norway	1
Sweden	2
7 countries	18
