# Supplementary material for: Precision phenotyping of a barley diversity set reveals distinct drought response strategies
Source: Front Plant Sci. 2024 Jun 24;15:1393991. doi: 10.3389/fpls.2024.1393991 (PMC11231632; doi:10.3389/fpls.2024.1393991)
Supplement: Supplementary file 12 [file Table_3.pdf]

Table S3. Maximum transpiration rate TRmax uder well-watered conditions; critical SWC **θ**c during drought stress from 81-line screen

Group	Code	Name	TRmax	**θ**c
A	2024	Artturi	10,42	35,59
A	2087	Frisia	11,48	29,86
A	2073	Eero	12,68	30,22
B	2102	Herse	13,95	18,06
B	2051	Chanell	14,02	25,60
B	2079	Etu	15,79	25,18
B	2093	Hankkija_673	15,88	16,83
B	2082	Favorit	16,87	23,78
C	2086	Freja	18,34	28,97
C	2104	Isaria	18,51	27,83
C	2103	Hydrogen	18,79	28,01
C	2101	Gate	19,02	27,96
C	2097	Gorm	20,22	29,40
D	2025	Arvo	22,51	30,00
D	2037	Binder	26,20	30,00
D	2084	Formula	27,18	31,41
D	2033	Barke	28,01	30,00
D	2034	Baronesse	29,18	30,00
