# Supplementary material for: Precision phenotyping of a barley diversity set reveals distinct drought response strategies
Source: Front Plant Sci. 2024 Jun 24;15:1393991. doi: 10.3389/fpls.2024.1393991 (PMC11231632; doi:10.3389/fpls.2024.1393991)
Supplement: Supplementary file 13 [file Table_4.pdf]

Table S4. Maximum transpiration (Emax) under well-watered conditions; critical SWC **θ**c during drought stress, 18 lines

Group	Code	Name	**θ**c	Std. Error o	Emax	Std. Error of Mean
Group 1	2103	Hydrogen	42,6533	2,918	15,1384	1,29488
	2093	Hankkija_673	40,3231	2,27564	14,4747	0,94255
	2034	Baronesse	39,087	1,97292	13,7502	0,50675
	2104	Isaria	38,7049	1,50548	12,7957	0,58368
Group 2	2033	Barke	40,1149	2,31025	13,4909	0,62889
	2097	Gorm	35,8127	2,43632	13,1754	0,60869
	2101	Gate	36,4074	1,11428	12,5033	0,21707
	2051	Chanell	34,1508	1,42127	12,0246	0,55855
	2082	Favorit	34,5064	2,23584	11,8246	0,57105
	2087	Frisia	36,0204	2,58709	11,7394	0,90145
	2079	Etu	36,9027	0,52283	11,4477	0,57186
Group 3	2025	Arvo	35,1197	2,10051	12,7548	0,59304
	2037	Binder	31,5549	2,24836	11,1248	0,52813
	2084	Formula	30,0064	3,07542	10,952	1,87731
	2073	Eero	30,5805	2,18401	10,5412	1,57636
	2102	Herse	33,0353	1,79695	9,3969	0,64774
	2024	Artturi	31,4731	2,30717	9,2269	0,70764
	2086	Freja	26,7912	1,23907	8,8455	0,78473
