# Supplementary material for: Precision phenotyping of a barley diversity set reveals distinct drought response strategies
Source: Front Plant Sci. 2024 Jun 24;15:1393991. doi: 10.3389/fpls.2024.1393991 (PMC11231632; doi:10.3389/fpls.2024.1393991)
Supplement: Supplementary file 14 [file Table_5.pdf]

Table S5. Summary of 18-line physiological strategy and performance

Lines	81-line grouping	18 line grouping	Drought Strategy	Vigor	Resilience	Recovery
Hankkija_673	B	1	dynamic	high	low	medium
Isaria	C	1	dynamic	high	medium	medium
Hydrogen	C	1	dynamic	high	medium	high
Baronesse	D	1	dynamic	high	medium	high
Frisia	A	2	isohydric	medium	medium	medium
Etu	B	2	isohydric	medium	low	low
Favorit	B	2	isohydric	low	medium	medium
Chanell	B	2	isohydric	medium	medium	low
Gate	C	2	isohydric	medium	medium	medium
Gorm	C	2	isohydric	medium	medium	high
Barke	D	2	isohydric	medium	medium	medium
Eero	A	3	anisohydric	low	medium	medium
Artturi	A	3	anisohydric	medium	low	medium
Herse	B	3	anisohydric	medium	low	low
Freja	C	3	anisohydric	low	high	high
Arvo	D	3	anisohydric	medium	medium	medium
Binder	D	3	anisohydric	low	high	high
Formula	D	3	anisohydric	low	high	high
